# Spinal Tuberculosis – Directly Observed Treatment and Short Course or Daily Anti Tubercular Therapy -Are We Over Treating?

**DOI:** 10.2174/1874325001812010380

**Published:** 2018-09-28

**Authors:** Ravinder Kumar Banga, Jagdeep Singh, Anshul Dahuja, Radhe Shyam Garg

**Affiliations:** 1 Department of Orthopaedics, Government Medical College, Patiala, Punjab, India; 2 Department of orthopaedics, Guru Gobind Singh Medical College and Hospital, Faridkot, Punjab, India

**Keywords:** ATT, DOTS, Spinal Tuberculosis, Erythrocyte Sedimentation Rate values (ESR), Visual Analogue Scale (VAS), Therapy

## Abstract

**Study Design:**

Prospective randomised control trial.

**Purpose:**

To compare the effectiveness of Directly Observed Treatment and Short Course (DOTS) and Daily Anti Tubercular Therapy (ATT) in spinal tuberculosis with no neurological deficit.

**Overview of Literature:**

Spinal tuberculosis is rampant in India with a major cause of morbidity and mortality. There is a lot of conflict regarding management with anti tubercular drugs, the regimen to be followed and the duration the drugs needed for complete cure.

**Material and Methods:**

This prospective study was conducted during the period of 2006 to 2009. Thirty cases of spinal tuberculosis were randomly divided equally into two groups of fifteen each and treated with DOTS and Daily ATT and compared at the end of follow up on clinical, radiological and Erythrocyte Sedimentation Rate values (ESR).

**Results:**

Pain score on Visual Analogue Scale (VAS) exhibits that mean pain score was 5.93+_1.54 at start and 0.64+_1.01 at the end of follow up with 89.39% change with DOTS therapy whereas mean pain score was 7.08 +_1.61at start of therapy and 0.69+_0.95 at end of follow up with 91.73% change in patients treated with Daily ATT with *p* value >0.05 (not significant). The radiological recovery in patients with DOTS and Daily ATT have similar results after 1.5 years with p value > 0.05 showing that the radiological recovery by both treatment modalities are comparable. Mean change in ESR scores in DOTS therapy patients and patients on daily ATT were 74.57+_9.34% and 75.69+_9.38% change with p value >0.05 which was not significant.

**Conclusion:**

DOTS therapy is an effective means of management of spinal tuberculosis cases with no neurological involvement, however its efficacy in patients with neurological deficit is further to be evaluated.

## 1 INTRODUCTION

Tuberculosis (TB) is a major cause of suffering and death since times immemorial. Though mycobacterium was discovered more than 100 years ago but it still continues to be a big health hazard even today despite the development of highly effective drugs for cure [1-3]. more than 1,000 people a day, (one every minute) die of tb in our country. India accounts for 1/5th of global incidence of TB and tops the list of 22 top TB burdened countries. Tuberculosis of bone and joints is rampant in India with the Dorso lumbar spine as the most common site of osseous involvement constituting 50% of osteoarticular tubercular cases. The management of spinal tuberculosis has evolved over time from chemotherapy alone, radical surgery combined with drugs, to the present times in which most of the patients are managed with chemotherapy and surgical intervention is done in cases with specific indications like no response to chemotherapy, significant or worsening neurological status, kyphosis, and abscess [4]. Efficacy of treatment depends on the drug regimen, drug combination and number of drugs used, duration of therapy, frequency of drug administration along with compliance as drug resistance is one of the major problems in india. The optimum duration of treatment was unresolved between 6 months 9 months, 12 to18 months or more based on evidence of clinic radiological healing. At present, World Health Organisation (WHO) recommends Directly Observed Treatment and Short Course therapy (DOTS) Category1 for all cases of extra pulmonary skeletal tuberculosis [5]. The DOTS regimen is based on intermittent drug intake and short course chemotherapy which is under direct supervision for better drug compliance. However, there is paucity of data which showed effectiveness of DOTS in spinal tuberculosis and because of fear failure of treatment due to short duration of therapy has constrained the surgeons not to abandon the practice of using daily regime of Anti Tubercular Therapy (ATT). So in this study, we compared the efficacy of daily and DOTS therapy in spinal tuberculosis.

## 2 MATERIALS AND METHODS

**Fig. (1):**
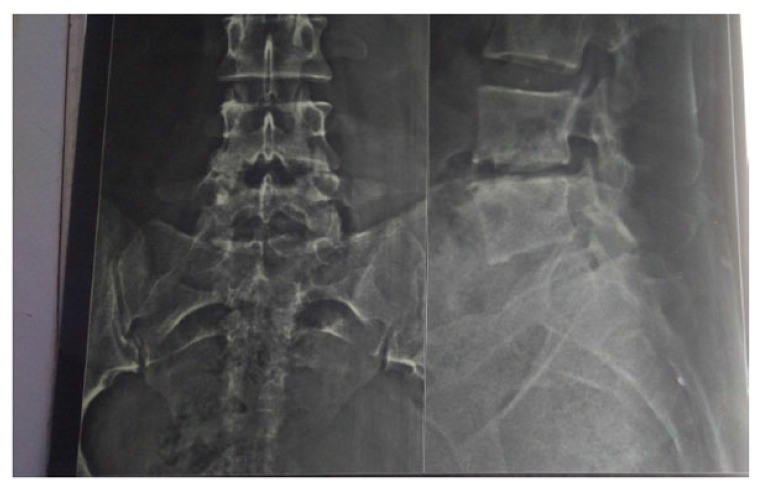
Skiagram AP and Lateral view of Lumbosacral spine with diminished intervertebral disc space and erosion of inferior and superior end plate of L4-L5vertebra.

**Fig. (2):**
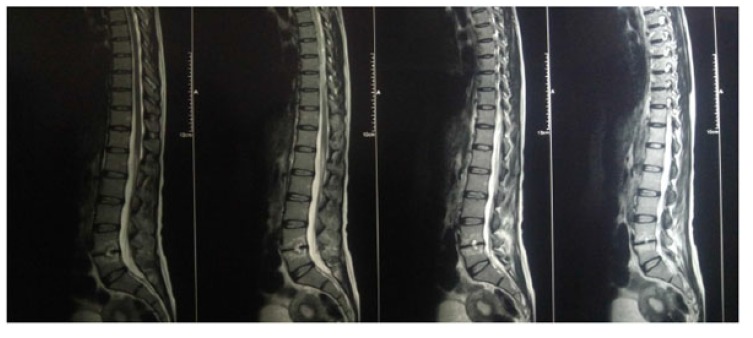
MRI lumbo sacral spine sagittal view showing reduced height of L4-L5 vertebrae with altered marrow signal intensity and irregularities in end plates. T2 weighted images .

**Fig. (3):**
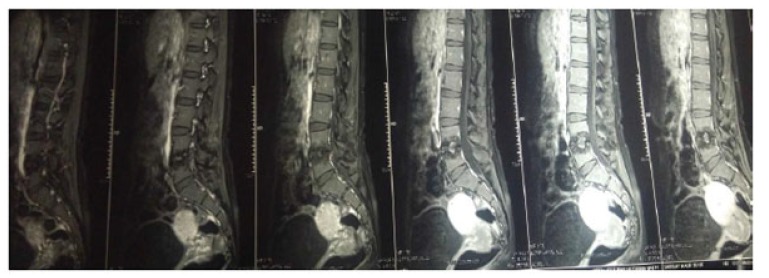
MRI sagittal section T1 post contrast image depicts significant heterogeneous enhancement at L4 -L5level.

The present prospective study was carried out in the department of author institute during the period of June 2006 to May 2009. Thirty patients of spinal tuberculosis were selected at random and distributed randomly into two groups of fifteen each (group A and group B). The new cases of Pott’s spine attending the Orthopaedic Outpatient Department (OPD) or emergency who were diagnosed on the basis of clinical examination, radiological assessment, Erythrocyte Sedimentation Rate (ESR), biopsy and with no neurological deficit were included in the study. The clinical features like persistent localized back pain, constitutional symptoms such as loss of weight, loss of appetite and evening rise of temperature, physical signs like localized tenderness, reduced range of movement, cold abscess, spinal deformity were taken in consideration. The radiological diagnosis was made on observation of demineralization of the vertebra, fuzzy paradiscal margin, reduction/obliteration of disc space, along with one or more of the following: destruction of end plates, wedging of vertebra, obvious kyphotic deformity, paravertebral shadows, and anterior scalloping of vertebral body (Fig. **1**) [6, 7]. On Magnetic Resonance Imaging (MRI), the characteristic picture comprised of marrow edema of vertebral body with end plate erosions and discitis along with pre- and paravertebral septate loculated collection including intraosseous abscess with subligamentous collection and epidural extension (Figs. **2**, **3**) [6-8]. The cases who had taken anti tubercular treatment in the past, skip lesion TB, TB meningitis and human immunodeficiency virus (HIV) positive patients were excluded from the study.

Group A patients were treated on category I DOTS therapy in which the patients were administered 2(HRZE)3 4 (HR)3 regimen for six months [9] H: Isoniazid (600 mg), R: Rifampicin (450 mg), Z: Pyrazinamide (1500 mg), E: Ethambutol (1200 mg). Patients who weighed more than 60 kg receive additional Rifampicin 150 mg. The drugs were administered three times a week under direct supervision.

The patients of Group B were treated by daily anti tubercular drug administration in the doses of Isoniazid (300mg), Rifampicin (450 mg) Pyrazinamide (1500mg), Ethambutol (800mg) .the patients were administered four drugs (HRZE) for first 3 months followed by two drugs (HR) for nine months and were followed up monthly.

The patients of spinal tuberculosis were advised bed rest for 6 to 8 weeks till pain subsided and later were made ambulatory by braces, however if patients were having progression of neurological deficit or appearance of neurological deficit after three weeks of anti tubercular therapy surgical intervention was done.

Follow up indoor patients were observed weekly and earlier in some cases to see improvement of patients regarding pain, clinico-radiological, blood parameters. The patients were advised to follow up monthly after discharge and followed upto 15 months and clinical, radiological status and ESR were recorded. The patients were assessed on the following variables:

### 1) Clinical assessment:

***a) Pain:*** on *visual analogue scale* – a 10 cm line was used with 0 labelled with no pain and 10 with worse pain. The line was marked at a point corresponding to the assessment of pain. The distance from 0 to 10 was be measured.

### 2) Radiological assessment:

**Fig. (4):**
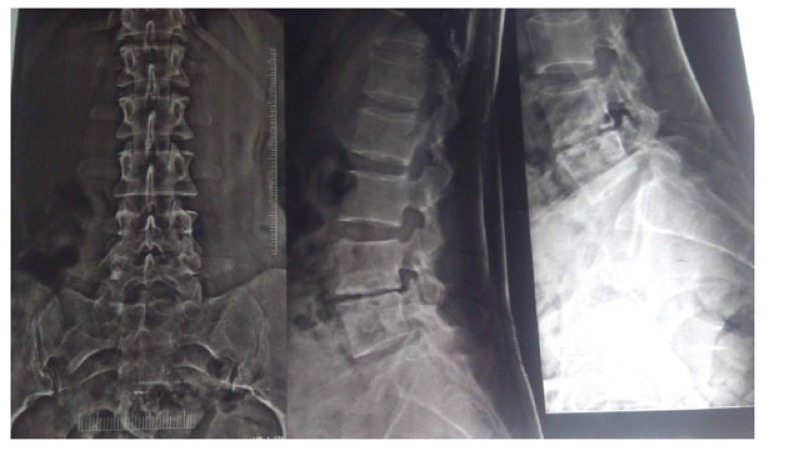
Shows x-ray of same patient of Lumbosacral spine AP and Lateral view demonstrates and reduction of end plate irregularity suggestive of healing.

**Fig. (5):**
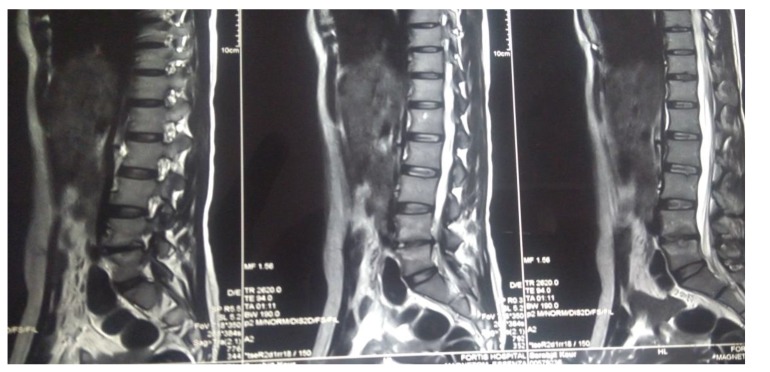
MRI lumbosacral spine sagittal view T2 weighted sequence of same patient showing near normalization of marrow signal.

Radiological signs of healing by means of x-ray, *i.e*., cessation of bone destruction, appearance of the normal trabecular pattern, correction of osteopenia, bone block or bony ankylosis, and regression in the size of the abscess were assessed (Fig. **4**). MRI criteria for healed status considered are complete resolution of pre- and para vertebral abscess, replacement of marrow oedema of the Vertebral body by fat or calcification (Fig. **5**). The patients were assessed radio logically by x-ray examination monthly and MRI examination six monthly and were graded as follows:

- Active
- Healing
- Healed
- Deteriorating

### 3) ESR:

ESR was done monthly and was recorded and assessed as
  - Increased
  - No change
  - Decreased

All the variables in both the groups were assessed and results evaluated.

Statistical analysis: The continuous variables were declared as mean ± Standard Deviation (SD). Depending on the distribution, mean values were compared using student t test. Statistical significance was determined with P <.05.

## 3 RESULTS

Thirty patients of spinal tuberculosis were taken up for the study from December 2006 to November 2009. Of them 27 patients were included in the study. Three patients were excluded because of loss of follow up (n=3). Majority of the patients had dorsal spine involvement *i.e* 14 out of 27 patients (51.8%). Details of the distribution were shown in (Table **1**). Most of the cases were in the age group of 21-40 years *i.e* 14 out of 27(51.8%) as shown in (Table **2**). Pain was the most common symptom and it was present in all the patients and next common clinical features was deformity. 8 patients out 27 had abscesses with sinus formation and in three patients had associated pulmonary tuberculosis as shown in (Table **3**). Pain score on visual analogue scale exhibits that mean pain score was 5.93+_1.54 at start of therapy and 0.64+_1.01 at end of therapy with 89.39% change in spinal tuberculosis treated with DOTS therapy whereas mean pain score was7.08 +_1.61at start of therapy and 0.69+_0.95 at end of therapy with 91.73% change in patients treated with daily anti tubercular therapy with *p* value >0.05 showing that the result is not significant. These values show that both the treatment modalities have similar pain relief at end of therapy and are comparable.

The radiological recovery in patients of spinal tuberculosis with DOTS and daily ATT therapy have similar results after one year (Table **4**). The x2 value of 0.541 and *p* value > 0.05 showing that the radiological recovery by both treatment modalities are comparable.

The comparison of ESR score in spinal tuberculosis exhibits that mean ESR score was 86.57+_15.98 at the start of therapy and 21.54+_6.79 at the end of therapy with 74.57+_9.34% change in patients treated with dots therapy and that mean ESR score was 89.57+_17.10 at start of therapy and 20.93+_7.15 at end of therapy with75.69+_9.38% change in patients treated with daily anti tubercular therapy with *p* value >0.05 showing that there is not a significant difference in the ESR fall in both treatment modalities . These values show that both the treatment modalities have similar ESR decrease at end of therapy and are comparable and equally effective.

## 4 DISCUSSION

In pre anti tubercular era nearly 75% of patients when treated in sanatorium, sanatorium treatment regimes which includes rest, fresh air, sunshine rooftops and patients used to die or were severely crippled from further complications of disease [10]. Introduction of modern anti-tubercular drugs and newer imaging modalities has enabled the physician and surgeons to make early diagnosis of disease and early start of treatment with ATT in pre-destructive stage has reduced the sequelae of neurological deficit and kyphotic deformity. Effectiveness of dots therapy in pulmonary and extra pulmonary case apart from skeletal ones is well established in the present era [11]. Dutt AK and Moer D have also advocated the use of short course chemotherapy with similar results in 95% cases of extra pulmonary tuberculosis [12]. Cohn DL, Catlin BJ and Peterson KL have results in which twice weekly treatment regimen was found to be efficacious and relatively non toxic especially in patients with dots therapy [13]. Venugopal k, Sreelatha PR, Philip S, Kumar V reported good results with DOTS therapy in neurotuberculosis [14].

The success of shorter intermittent therapy regimen is due to better patient compliance, advantage of being less toxic, availability of drugs at free of cost under revised national tuberculosis control programme (RNTCP)program and more patient contact with the drug provider ensuring drug intake. However, its role in spinal tuberculosis is not well defined. Rational behind the use of DOTS regime in skeletal tuberculosis was formed by studies at Tuberculosis Research Centre (TRC), Chennai, India, have shown that twice-weekly regimen was as effective as the daily-unsupervised regimen [15]. But majority of orthopaedic surgeon are still in favour of using daily regime of ATT because of paucity of relevant data on efficacy of intermittent therapy. Moreover, the consensus over the proper duration of therapy as end point of disease is not well established [16]. There is insufficient data to support the role of short course intermittent chemotherapy in TB of spine. Balasubramaniam R and Ramachandram S [17] have advocated that short course chemotherapeutic regimens have been proven to be highly effective in tuberculosis of spine, and Pott’s paraplegia and that the intermittent regimens have been found to be as effective as daily regimen. Rajeshwari R, Balasubramaniam R and Venkatesan Pin their study have concluded that a combination of surgery and short course chemotherapy of 9 month duration is effective in treatment of Pott’s paraplegia [4]].Medical research council working party on tuberculosis of the spine in their study have pointed that 6 and 9 month regimen were equally effective as 18 months regimen and chemotherapy is a critical factor in management of tuberculosis of spine [18]. A prospective clinical study was conducted on a consecutive series of patients with spinal tuberculosis treated by category I (RNTCP) regimen based on DOTS strategy of WHO from 2004 to 2007 to evaluate the efficacy and concluded that efficacy of DOTS was comparable with other standard regimens . It also described significant reduction in adverse side effects when compared with daily regimens with better outcome in those treated early [19].Results of our study in terms of clinico- radiological and blood investigation criteria showed no significant difference between 6 months DOTS therapy and 12 months daily ATT regime with p value >.005. Wang Z, Ge Z, Jin W *et al* [20] in their study have concluded that no significant differences in clinical cure rates with 41/2, 8 and 12 months therapy and had lower adverse effects with shorter chemotherapy durations .

The literature is replete in terms of exact duration of treatment in musculoskeletal TB. Tuli's middle-path regimen is widely used with good results [21]. The British Thoracic Society recommends 6 months' duration for spine unless there is central nervous system involvement where they recommend 12 months therapy [22]. Upadyay SS, Saji M and Yau A Cin their study in cases of spinal tuberculosis have reported comparable radiological results with 6, 9 and 18 months of chemotherapy [23]. A recent study was conducted to evaluate the efficacy of extended DOTS regimen (2 months of intensive phase and 6 months of continuation phase) as recommended by WHO, by using MRI observations as the healing marker which concluded that 60% of patients had shown healed status, who had completed 12 months of DOTS category- I therapy in spite of clinical and hematological improvement and 90% patients showed healed status on MRI at 18 months [16]. This study emphasized that extended DOTS is an effective therapy, however to document the healing status, spinal lesions should be evaluated clinically, hematologically, radiologically, and by contrast MRI and suggested that not to stop ATT by fixed time frame after 8 months of ATT.

Limitation of study is less number of patients in both the groups with shorter follow-up. This study does not evaluate the results in terms of side effects of both types of regime. Another limitation is we did not include the patient with neurological deficit in this study.

Future directions: So, further studies should be done to compare the efficacy of DOTS versus daily regime with larger group of patients with longer follow up which also includes improvement in neurological status criteria along with side effects caused by these two therapies.

## SUMMARY AND CONCLUSION

We observed that the results are comparable at one year of follow up on clinical, radiological and ESR values. The patients on both DOTS and daily ATT therapies had good response to treatment and we had no relapse during the follow up. Thus, we conclude that DOTS therapy is an effective means of management of spinal tuberculosis in cases with no neurological involvement with advantage of lesser side effects, better compliance and free therapy in developing countries decreasing financial load on the patients but its role in more severe cases with neurological deficit is further to be evaluated where the duration needs to be titrated as per patients needs.

## Figures and Tables

**Table 1:** Showing regional distribution of cases.

**SPINAL TUBERCULOSIS** **(27 CASES)**	**REGION**	**NO. OF PTS**
	**DORSAL**	**14**
	**LUMBAR**	**8**
	**DORSOLUMBAR**	**3**
	**CERVICAL**	**2**

**Table 2:** Showing age group and sex distibution of cases.

	**AGE GROUP(YEARS)**	**NO. OF PTS**		**TOTAL**
		**MALES**	**FEMALES**	
	0-20	2	1	3
	21-40	8	6	14
	>40	5	5	10
**TOTAL**		15	12	27

**Table 3:** Showing clinical features.

**FEATURES**	**DORSAL**	**LUMBAR**	**DORSOLUMBAR**	**CERVICAL**	**TOTAL**
**PAIN/TENDERNESS**	14	8	3	2	27
**ABSCESS**	4	2	2	0	8
**SINUS**	2	0	1	0	3
**DEFORMITY**	7	5	2	0	14
**ASS. PUL. TB**	1	1	1	0	3

**Table 4:** Showing comparison of radiological recovery in daily and dots att at he end of 15 months followup.

	**SPINAL TB DOTS**	**SPINAL TB DAILY**	**P Value**	**Significance**
**Healed**	12	13	0.462	Not Significant
**Healing**	1	1		
